# A comprehensive examination of temporal-seasonal variations of PM_1.0_ and PM_2.5_ in taiwan before and during the COVID-19 lockdown

**DOI:** 10.1007/s11356-024-33174-4

**Published:** 2024-04-18

**Authors:** Shahzada Amani Room, Yi Chen Chiu, Shih Yu Pan, Yu-Cheng Chen, Ta-Chih Hsiao, Charles C.-K. Chou, Majid Hussain, Kai Hsien Chi

**Affiliations:** 1 Institute of Environmental and Occupational Health Sciences, National Yang Ming Chiao Tung University, Taipei, 112 Taiwan; 2 National Institute of Environmental Health Sciences, National Health Research Institutes, 35 Keyan Road, Zhunan Town, Miaoli, Taiwan; 3 Graduate Institute of Environmental Engineering, National Taiwan University, Taipei, Taiwan; 4 Research Center for Environmental Changes, Academia Sinica, Taipei, 115 Taiwan; 5 Department of Forestry and Wildlife Management, University of Haripur, 22620, Hattar Road, Haripur City, KP Pakistan

**Keywords:** COVID-19, Taiwan, Health risk, PM_1.0_, PM_2.5_, PAHs

## Abstract

COVID-19 has been a significant global concern due to its contagious nature. In May 2021, Taiwan experienced a severe outbreak, leading the government to enforce strict Pandemic Alert Level 3 restrictions in order to curtail its spread. Although previous studies in Taiwan have examined the effects of these measures on air quality, further research is required to compare different time periods and assess the health implications of reducing particulate matter during the Level 3 lockdown. Herein, we analyzed the mass concentrations, chemical compositions, seasonal variations, sources, and potential health risks of PM_1.0_ and PM_2.5_ in Central Taiwan before and during the Level 3 lockdown. As a result, coal-fired boilers (47%) and traffic emissions (53%) were identified as the predominant sources of polycyclic aromatic hydrocarbons (PAHs) in PM_1.0_, while in PM_2.5_, the dominant sources of PAHs were coal-fired boilers (28%), traffic emissions (50%), and iron and steel sinter plants (22.1%). Before the pandemic, a greater value of 20.9 ± 6.92 μg/m^3^ was observed for PM_2.5_, which decreased to 15.3 ± 2.51 μg/m^3^ during the pandemic due to a reduction in industrial and anthropogenic emissions. Additionally, prior to the pandemic, PM_1.0_ had a contribution rate of 79% to PM_2.5_, which changed to 89% during the pandemic. Similarly, BaPeq values in PM_2.5_ exhibited a comparable trend, with PM_1.0_ contributing 86% and 65% respectively. In both periods, the OC/EC ratios for PM_1.0_ and PM_2.5_ were above 2, due to secondary organic compounds. The incremental lifetime cancer risk (ILCR) of PAHs in PM_2.5_ decreased by 4.03 × 10^-5^ during the pandemic, with PM_1.0_ contributing 73% due to reduced anthropogenic activities.

**Supplementary Information:**

The online version contains supplementary material available at 10.1007/s11356-024-33174-4.

## Introduction

The corona virus (COVID-19) has been a major global issue due to its contagious nature (Arregocés et al. 2021). In December 2019, an outbreak of this virus was reported in Wuhan, China (Hua and Shaw 2020), and has since spread both domestically and internationally as of 30 January 2020 (Mishra and Mishra 2021). Consequently, the World Health Organization to declare it a global public health emergency on March 11, 2020 (Jandrić 2020). Afterward, a series of control measures were put in place and many countries have imposed national or partial closures, as well as restrictions on human activities, to combat the disease (Dantas et al. 2020). Interestingly, these restrictions have significantly contributed to the reduction of global air pollution (Venter et al. 2020). Previously (Brugge et al. 2007; Sharma et al. 2020; Xie and Zhu 2020) has shown a remarkable decline in fine aerosols during the global COVID-19 lockdowns. For example, 20–60% reduction in PM_2.5_ in Malaysia (Nadzir et al. 2020), 69.46% in India (Fatima et al. 2022), 26% in Croatia (Jakovljević et al. 2021), and 50% off PM_1.0_ in China (Xu et al. 2020), respectively. Like other countries, Taiwan has also strived to address the global pandemic of COVID-19 (Wu et al. 2022a, 2022b). The first case of COVID-19 in Taiwan was reported on 21 January 2020 (Cheng et al. 2020). Since then, the Taiwanese government has implemented various measures to prevent the spread of the disease, for example mask-wearing, social distancing, travel restrictions, and school closures (Yu et al. 2021). Additionally, Taiwan's National Health Command Center (NHCC) declared a Level 3 COVID-19 Alert from 19 May to 27 July 2021 due to a rise in locally transmitted cases (Wu et al. 2022a, 2022b).

In recent decades, fine particulate matter (PM_1.0_ and PM_2.5_) have become a global health concern due to their small size and hazardous effects (Song et al. 2022). PM_1.0_ is one of the significant components of PM_2.5_ and is considered more significant due to its high toxicity (Liu et al. 2022). PM_2.5_ is typically caused by coal combustion, biomass burning, vehicular emissions, and energy production process (Fatima et al. 2022). Recent epidemiological studies (Sun et al. 2015; Dong et al. 2018) have confirmed a linked between high levels of PM_2.5_ and increased morbidity and mortality. It is estimated that 0.31 and 7.02 million deaths from global cardiovascular and lung diseases were attributed to PM_2.5_ (Yang et al. 2022). Likewise, 4.1 million people died worldwide in 2016 due to ambient PM_2.5_ (Gakidou et al. 2017). In Taiwan, PM_2.5_ is a significant concern (Chang and Lee 2008), as it has been shown to have adverse effects on respiratory and cardiovascular health, resulting in increased mortality rates (Wang et al. 2021). Prior studies in Taiwan have examined the effect of control measures on air quality during the pandemic. However, further research is needed to compare various time periods and evaluate the health consequences associated to the decrease in particulate matter concentrations resulting from the Level 3 lockdown. Hence, this study aims to examine mass concentrations, chemical compositions, seasonal variations, potential sources, and associated health risks of PM_1.0_ and PM_2.5_ in Central Taiwan, both before and during the pandemic Level 3 lockdown in 2021. We hope that our findings will encourage policymakers to enhance air quality in the proposed area and also serve as a valuable resource for cities in other countries.

## Material and methods

### Description of the sampling location and collection

**Fig. 1:**
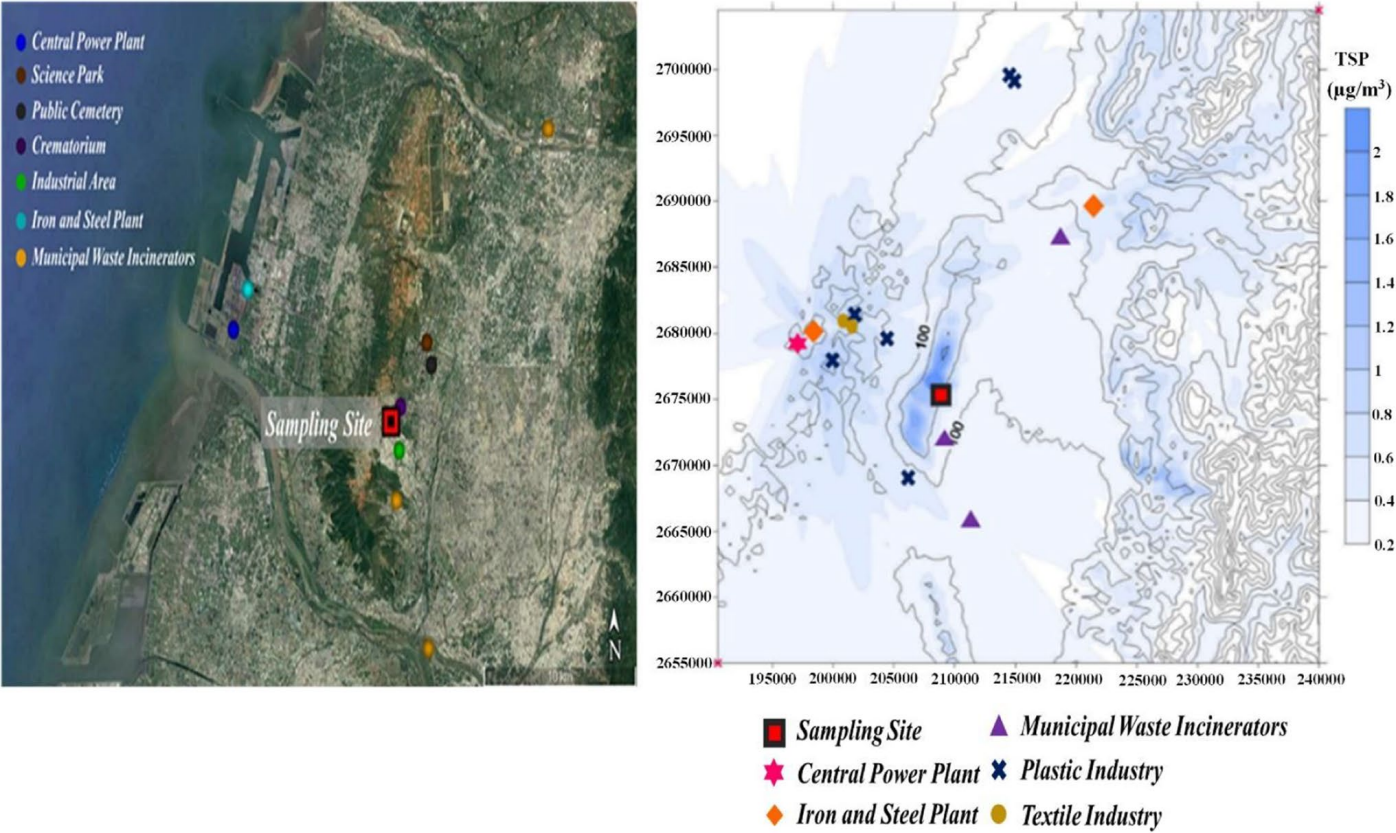
Sampling site and AERMOD simulation analysis

Taichung city has been selected as the study area. As the second most populated city, it boasts a population density of 2.81 million (Su et al. 2021). It is a major industrial and transportation hub, spanning an area of 2214 km^2^ (coordinates-latitudes: 24°10'55.0"N, longitude: 120°35'45.4"E) (Hung and Lee 2021) (Fig. 1). However, due to its rapid population growth coupled with industrial expansion, Taichung's air quality has been adversely impacted by fine particles originating both locally and from Taiwan’s northern and southern counties (Fang and Zhuang 2022).

**Fig. 2:**
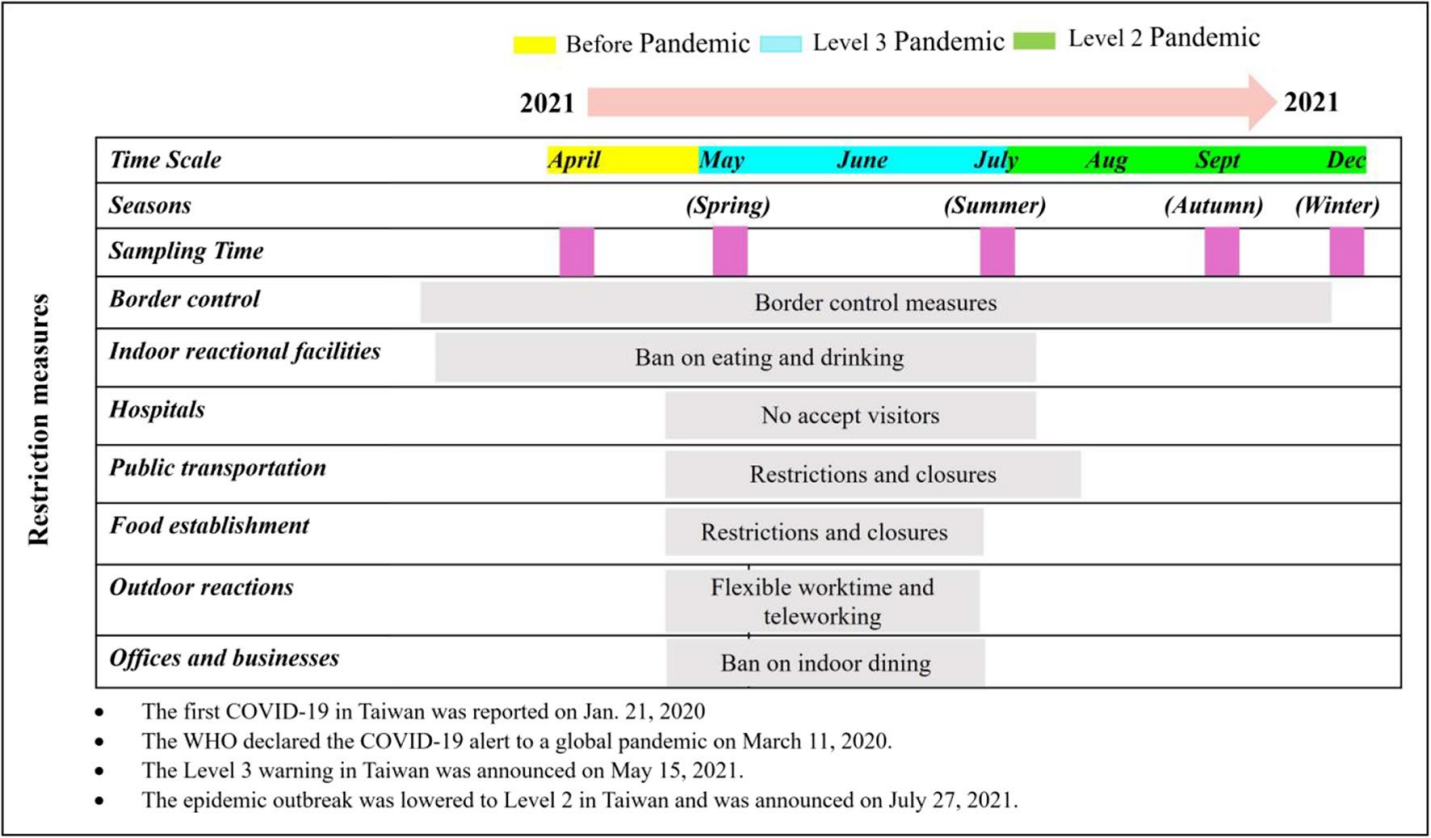
Timeline of the COVID-19 Pandemic's restriction measures

In this study, PM_1.0_ (n=54) and PM_2.5_ (n=54) samples were collected simultaneously on circular quartz fiber filter (150 mm diameter) at Tunghai University (coordinates-latitudes: 24°10'41"N, longitude: 120°36'13"E) in Taichung, using a DHA-80 (Digitel, Switzerland) high volume air sampler (HVS) operating at a flow rate of 500 L/min. There were three sampling campaigns, such as pre-pandemic alert (3-15 April 2021), during the pandemic alert level 3 (PAL3) (11-23 May 2021 and 1-6 July 2021), and during the pandemic alert level 2 (PAL2) (4-9 September 2021 and 3-8 December 2021) as illustrated in Fig. 2. Data collection lasted 24 hours in April and May for one sample each, while during Summer, Autumn, and Winter, the sampling periods were 12 hours due to dust loading on filters. Moreover, during sampling, meteorological data including wind direction, temperature, etc. were sampled from the Environmental Protection Agency (Taiwan), and atmospheric pollutants such as SO_2_, O_3_, NO_2_, PM_1.0_ and PM_2.5_ were obtained from Taichung’s Environmental Monitoring Station near the sampling site. Also, AERMOD was used to simulate ambient sampling stations. Furthermore, the study utilized both Potential Source Contribution Function (PSCF) and Positive Matrix Factorization (PMF) methodologies to accurately detect sources contributing to air pollution at the sampling location.

### Sample analysis

Primarily, the filters were dried at 500°C for 24 hours, then wrapped in aluminum foil and stored in a room with a temperature of 20 ± 2°C and humidity of 45% ± 5%. Afterwards, they were promptly sealed and kept in a refrigerator maintained at -18°C. To ascertain the mass value of fine particles, quartz filters were weighed three times using a micro analytical balance (CP225D, Sartorius, Germany) with a sensitivity of 0.1 mg, adhering to controlled conditions of 20 ± 2°C for temperature and 50 ± 5% humidity pre- and post-sampling, as detailed by (Liu et al. 2020). Additionally, blanks were taken for each of the six PM_1.0_ and PM_2.5_ measurements to negate any potential pre- and post-sampling biases during filter handling (Liu and Corma 2018). Besides, different instruments were utilized in this research to analyze the chemical composition of PM_1.0_ and PM_2.5._ By utilizing (GC MS/MS, Thermo Fisher Scientific Taiwan Co., Ltd., Taiwan) (recovery range; 80% to 120% and detection limits; 0.30 to 2.1 ng), a total of 27 PAHs were examined. In addition, the DionexTM ICS-1000 (Thermo Fisher Scientific Inc., Waltham, MA, USA) was used to analyze ten water-soluble ions including NO_3_^-^, Cl^-^, PO_4_^3-^, SO_4_^2-^, Na^+^, NO_2_^-^, NH_4_^+^, Mg^2+^, Ca^2+^ and K^+^ (recovery range; 88% to 104% and detection limit; 0.01 to 10.1 ng). As well, the OC and EC were analyzed using the IMPROVE Thermo-Optical Reflection (TOR) method as outlined by Fung et al. (2002) and Chow et al. (2001). Additionally, the average recoveries for all species were found to fall within the range of 100 ± 5%. For further information regarding the sampling analysis, please refer to supplementary data text S1.

### Source apportionment

#### Positive Matrix Factorization (PMF)

The PMF (5.0 model) was utilized to evaluate the sources of PAHs in Taichung, known for its effectiveness in air quality assessment and source identification (Jindamanee et al. 2020). Widely adopted in analytical studies (Chang et al. 2018), further insights can be gleaned from related literature (Zhang et al. 2018; Park et al. 2019). Eq. (1) outlines the calculation methodology.

1 $${{x}}_{{ij}}=\sum_{{k}={1}}^{{p}}{{g}}_{{ik}}{{f}}_{{k}{j}}+{{e}}_{{ij}}\left({i}={1},{2},\cdots {n};{j}={1},{2}\dots {m};{k}={1},{2},\dots {p}\right)$$

where *x* includes *ⅈ* samples and *j* compounds, *j* is the species, *k* is the number of pollution sources, *g*_*ik*_ is the contribution of the *k*^th^ source to the *ⅈ*^th^ sample, *f*_*k*j_ is the profile of species *j* by the *k*^th^ source, and *e*_*ij*_ is the residual of the *ⅈ*^th^ sample and the *j*^th^ species concentration value and its analytical value. Uncertainties are handled differently based on whether the levels of chemical components surpass the detection limit (MDL) or not. When concentrations are less than or equal to the MDL, uncertainties are computed using Unc = 5/6 × MDL (Wang et al. 2022); else, they are determined using eq. (2).

2 $$\textbf{Error}={\left[\ {\left(\textbf{fraction}\times \textbf{concentration}\right)}^{\textbf{2}}+{\left(\textbf{0.5}\times \textbf{MDL}\right)}^{\textbf{2}}\right]}^{\textbf{1}/\textbf{2}}$$

#### Potential source contribution function

In this study, the PSCF method was used to recognize potential sources of PM_1.0_ and PM_2.5_, along with their chemical components, near the sampling location. This is a qualitative technique for ascertaining the pollutants sources in a specified location (Zhang et al. 2018). Detailed information can be obtained elsewhere (Petroselli et al. 2018). To compute PSCF values, the trajectory's geographical extent is partitioned into grid cells of variable sizes, determined by the back trajectory domain (Li et al. 2020). Its calculation method is given in eq. (3).

3 $${\boldsymbol{PSCF}}_{\boldsymbol{ij}}=\frac{{\boldsymbol{m}}_{\boldsymbol{ij}}}{{\boldsymbol{n}}_{\boldsymbol{ij}}}$$

Where *m*_*ij*_ is the number of segments and points in the air parcel trajectory combined with pollutant data in the *ij*^th^ grid and *n*_*ij*_ is the number of segments or points of air parcel in the *ij*^th^ grid.

### Health risk assessment

The study incorporated the ILCR to analyze potential health risks associated with ∑_27_ PAHs before and during the pandemic. Moreover, the toxicity values for each species are shown in Table S1. Typically, BaPeq values have been used to estimate the potential health risks linked with exposure to toxic PAHs (Liu et al. 2022), and its concentration is determined by multiplying each PAH concentration with its corresponding (BaP_TEQ) value, as illustrated in eq. (4). Subsequently, the calculated (BaP_TEQ) was multiplied by the unit risk (UR) to determine the ILCR as given in eq. (5).

4 $$\boldsymbol{BaP}\_\boldsymbol{TE}\boldsymbol{Q}={\sum}_{\boldsymbol{i}=\textbf{1}}^{\boldsymbol{n}}\left({\boldsymbol{C}}_{\boldsymbol{i}}\times \boldsymbol{BaP}\_\boldsymbol{TE}{\boldsymbol{F}}_{\boldsymbol{i}}\right)$$

5 $$\boldsymbol{ILCR}=\boldsymbol{U}{\boldsymbol{R}}_{\boldsymbol{BaP}}\times \boldsymbol{BaP}\_\boldsymbol{TEQ}$$

Where *BaP* _ *TEQ* is the toxic equivalent concentration of PAHs (ng/m^3^), *C*_*i*_ is the level of each PAHs species (ng/m^3^), and *BaP* _ *TEF*_*i*_ *is* the toxic equivalent factor of each species of PAHs, as for UR, is the unit risk (8.7 x 10^-5^ ng/m^3^).

## Results and discussion

### Comparing PM_1.0_ and PM_2.5_ concentrations in various scenarios

Table S2 displays the average concentrations of fine particles (PM_1.0_ and PM_2.5_) at various time intervals. The highest mean values of 16.6 ± 6.41 μg/m^3^ and 20.9 ± 6.92 μg/m^3^ were reported for both PM_1.0_ and PM_2.5_ at the study site before the pandemic. However, the lowest mean values of 13.0 ± 2.36 μg/m^3^ and 15.3 ± 2.51 μg/m^3^ were observed during the pandemic (PAL3). These measured levels are lower than those documented by Yu et al. (2021) for PM_2.5_ in northern and southern Taiwan. Prior to the pandemic, Taichung experienced elevated levels of fine particles, potentially caused by increased emissions from domestic sources. According to Chen et al. (2019), more than 60% of Taichung's air pollution is locally generated. Chen et al. (2022) revealed that approximately 42.7% of PM_2.5_ in Taichung is derived from industrial emissions, 31.9% from oil combustion, and 8.9% from traffic emissions. In addition, the sampling took place during the Qingming Festival, which typically occurs from 5-7 April each year. This festival involves cultural practices like grave cleaning, burning joss sticks/paper, and outdoor cooking, which could potentially lead to higher levels of ambient aerosols before the pandemic. In a previous study, Horng et al. (2007) observed a significant increase of 41.3 μg/m^3^ in PM_2.5_ levels at the study site during the Qingming Festival. Similar trends have also been observed in PM_2.5_ levels during the Spring Festivals in China (Chen et al. 2020; Zhang et al. 2023). In contrast, during PAL3, the Taiwanese government enforced rigorous control measures, including restrictions on anthropogenic activities, public gatherings, transportation, and the closure of markets, restaurants, industries, and universities (Chang et al. 2020; Yen et al. 2023). Interestingly, these measures potentially resulted in a decrease in PM_1.0_ and PM_2.5_ levels in the study area. Comparable observations have been documented elsewhere, indicating declines in fine aerosols during COVID-19 lockdowns (Bao and Zhang 2020; Manchanda et al. 2021; Xiong et al. 2021; Ivanovski et al. 2022).

Table S3 presents the fluctuations in mass concentrations, as well as the ratios of PM_1.0_ and PM_2.5_, across different seasons. The data reveals that PM_1.0_ significantly influences PM_2.5_ levels throughout all seasons, with a ratio higher than 0.5, aligning with Luo et al. (2022). Moreover, the highest mean values of fine particles were observed during winter (December; 19.7 ± 5.08 μg/m^3^ for PM_1.0_ and 27.1 ± 7.90 μg/m^3^ for PM_2.5_) and the lowest values were documented in spring (May; 13.0 ± 2.36 μg/m^3^ for PM_1.0_ and 15.3 ± 2.51 μg/m^3^ for PM_2.5_) whereas the concentrations during Summer (July) and autumn (September) were found to be intermediate, with values of 13.9 ± 3.10 μg/m^3^ and 17.8 ± 3.78 μg/m^3^ for PM_1.0_, and 15.7 ± 2.64 μg/m^3^ and 25.5 ± 2.42 μg/m^3^ for PM_2.5_, respectively. Several factors contributed to the observed results, including the implementation of Taiwan's Pandemic Alert Level 2 (PAL2) during the winter and autumn seasons, which led to certain changes in pandemic restrictions, such as the relaxation of quarantine and anti-epidemic measures for religious activities, food restaurants, supermarkets, entertainment venues, etc. (Yen et al. 2023). Additionally, during winter, the monsoonal winds facilitate the transport of pollutants from Mongolia and continental regions to Taiwan, contributing to an increase in atmospheric aerosols (Chi et al. 2017). Moreover, Taichung encounters high relative humidity (above 70%) and low wind speed (less than 0.1 m/s) during the winter season, which may also contribute to greater levels of ambient aerosols (Kuo et al. 2008; Hsu and Cheng 2019). Likewise, the relaxation of lockdown measures (PAL2) in autumn could lead to localized pollution, resulting in an increase in PM levels within the designated region. Similarly, dust storms from Mongolia and other continental areas during autumn contribute significantly to the elevated ambient air pollution (Chu et al. 2012). Conversely, summers in Taichung experience substantial rainfall, high humidity, and strong winds, which significantly reduce the amount of fine particles in the ambient air (Fang et al. 2000). It is worth noting that the enforcement of stringent measures during spring, prompted by PAL3, has led to a substantial reduction in PM_1.0_ and PM_2.5_ levels at the specified location.

Furthermore, the study examined ten water-soluble ions (WSIs), including PO_4_^3-^, Cl^-^, NO_2_^-^, SO_4_^2-^, Mg^2+^, Na^+^, Ca^2+^, NH_4_^+^, K^+^ and NO_3_^-^. To diminish the effects of marine aerosols on atmospheric ion concentration, we used the following eq: (6) to (9) (Room et al. 2023).

6 $$\left[\boldsymbol{nss}-\boldsymbol{S}{\boldsymbol{O}}_{{\textbf{4}}^{\textbf{2}-}}\right]=\left[\boldsymbol{S}{\boldsymbol{O}}_{{\textbf{4}}^{\textbf{2}-}}\ \right]-\textbf{0.25}\times \left[\boldsymbol{N}{\boldsymbol{a}}^{+}\right]$$

7 $$\left[\boldsymbol{nss}-{\boldsymbol{K}}^{+}\right]=\left[{\boldsymbol{K}}^{+}\right]-\textbf{0.038}\times \left[\boldsymbol{N}{\boldsymbol{a}}^{+}\right]$$

8 $$\left[\boldsymbol{nss}-\boldsymbol{C}{\boldsymbol{a}}^{\textbf{2}+}\right]=\left[\boldsymbol{C}{\boldsymbol{a}}^{\textbf{2}+}\right]-\textbf{0.038}\times \left[\boldsymbol{N}{\boldsymbol{a}}^{+}\right]$$

9 $$\left[\boldsymbol{nss}-\boldsymbol{M}{\boldsymbol{g}}^{\textbf{2}+}\right]=\left[\boldsymbol{M}{\boldsymbol{g}}^{\textbf{2}+}\right]-\textbf{0.12}\times \left[\boldsymbol{N}{\boldsymbol{a}}^{+}\right]$$

**Fig. 3:**
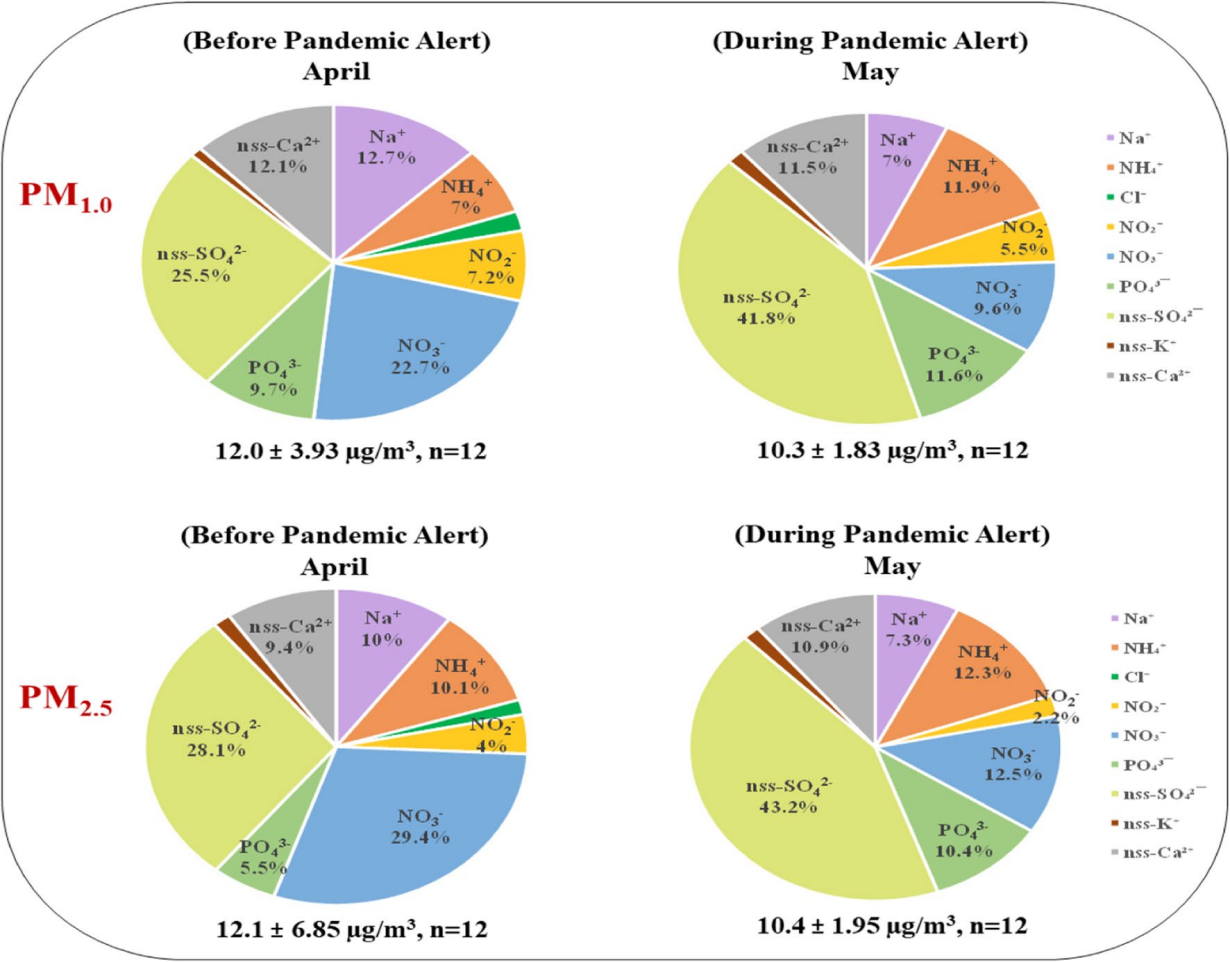
Distribution of water-soluble ions in PM_1.0_ and PM_2.5_ before and during the epidemic alert

The average concentrations of WSIs before and during the pandemic are given in Table S4. Before PAL3, the WSIs concentrations were 12.0 ± 3.93 μg/m^3^ for PM_1.0_ and 12.1 ± 6.85 μg/m^3^ for PM_2.5_ however, during PAL3, these values decreased to 10.3 ± 1.83 μg/m^3^ and 10.4 ± 1.95 μg/m^3^ respectively, as a result of reduced anthropogenic emissions. Prior to PAL3, PM_1.0_ primarily consisted of SO_4_^2-^, NO_3_^-^ and Na^+^ with respective contributions of 25.5%, 22.7%, and 12.7%. In contrast, PM_2.5_ was predominantly composed of SO_4_^2-^, NO_3_^-^ and NH_4_^+^ with contributions of 28.1%, 29.4%, and 10.1% as illustrated in Fig. 3. Tsai et al. (2016), categorize SO_4_^2-^, NO_3_^-^ and NH_4_^+^ as secondary inorganic aerosols (SIA). Notably, in this study, the SIA accounted for more than 50% for both PM_1.0_ and PM_2.5_, suggests that these ions are mainly released as secondary aerosols. It is noteworthy that there was a significant increase of 41.8% and 43.2% in SO_4_^2-^ in PM_1.0_ and PM_2.5_ during PAL3, suggesting the emission of secondary inorganic aerosols from coal combustion and other fossil fuels (particularly from power plants), which align with prior studies (Deshmukh et al. 2011; Satsangi et al. 2013; Liu et al. 2016). Remarkably, during PAL3, the NO_3_^-^ experienced a significant reduction (*p* > 0.05) with a decrease of 9.6% and 12.5% in PM_1.0_ and PM_2.5_ respectively. This decline can be attributed to a decrease in vehicle emissions, as observed in urban areas of China (Tian et al. 2021) and Spain (Clemente et al. 2022). Similarly, Xiong et al. (2021) also found a significant 66% decrease in NO_3_^-^ in China, mainly due to reduced private vehicle usage.

**Fig. 4:**
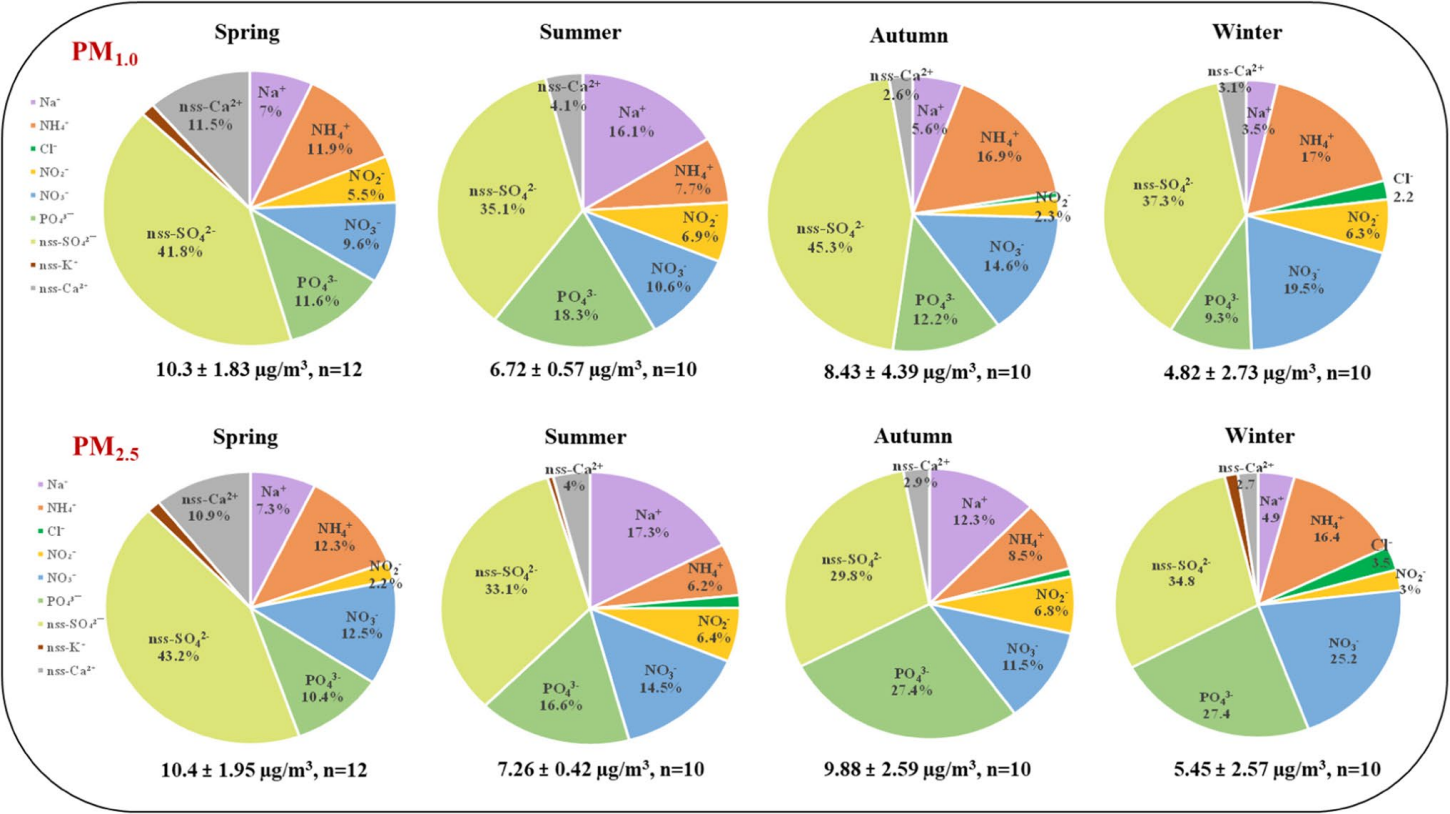
Distribution of water-soluble ions in PM_1.0_ and PM_2.5_ in different seasons

Table S5 demonstrates the seasonal fluctuations in WSIs prior to and during the COVID-19 lockdown. The PM_1.0_ to PM_2.5_ ratio surpasses 0.5 in different seasons, indicating that a considerable portion of the ions in PM_2.5_ likely originate from PM_1.0_, suggesting that these ions may have originated from anthropogenic sources (Pérez et al. 2008). Additionally, the main constituents of both PM_1.0_ and PM_2.5_ were found to be SO_4_^2-^, PO_4_^3-^, NO_3_^-^ and NH_4_^+^, exhibited significant fluctuations across different seasons, as shown in Fig. 4. According to Alastuey et al. (2006) and Wang et al. (2005), SO_4_^2-^, PO_4_^3-^ are associated with stationary emission sources, while NO_3_^-^ and NH_4_^+^ are linked to mobile emission sources. It is worth noting that the highest values of WSIs in both PM_1.0_ (10.3 ± 1.83 μg/m^3^, 8.43 ± 4.39 μg/m^3^) and PM_2.5_ (10.4 ± 1.95 μg/m^3^, 9.88 ± 2.59 μg/m^3^) were observed during the spring and autumn seasons, while the lowest values of (6.72 ± 0.57 μg/m^3^, 7.26 ± 0.42 μg/m^3^) and (4.82 ± 2.73 μg/m^3^, 5.45 ± 2.57 μg/m^3^) were documented in summer and winter (Table S5). This discrepancy may be caused by local pollution, as well as meteorological changes such as relative humidity, high wind speed and temperature (Yao et al. 2020). Unlike prior studies (Cheng et al. 2006; Wang et al. 2016), the reliability of our results is enhanced by the ongoing functioning of semiconductor industries, coal-power plants, food delivery services, and the long-range transportation of pollutants within the designated region during the spring. Also, the relaxation of PAL2 restrictions during autumn could lead to an increase in fuel combustion and anthropogenic emissions, potentially resulting in elevated ambient air pollution. Conversely, lower levels of WSIs during the summer may be indicative of higher wind speeds at the study site. Jiang et al. (2021) showed a notable decrease in different ion species as wind speed increased.

Table S6 presents the values of OC, EC, and OC/EC ratio for different time periods. The mean values of OC and EC were greater before the pandemic (PM_1.0_: 3.53 μg/m^3^, 1.05 μg/m^3^ and PM_2.5_: 3.79 μg/m^3^, 1.12 μg/m^3^) compared to during the pandemic (PM_1.0_: 1.74 μg/m^3^, 0.52 μg/m^3^ and PM_2.5_: 1.84 μg/m^3^, 0.53 μg/m^3^). Chang et al. (2020) and Sun et al. (2020) have also observed a linear pattern of OC and EC in China. EC is linked with primary combustion (Singh et al. 2017) however, OC can be derived from both primary and secondary organic compounds (Turpin and Huntzicker 1995; Huang et al. 2015). In the present study, it is interesting to note that during PAL3, there was a significant decrease in the levels of both OC and EC in PM_1.0_ and PM_2.5_, suggests that the preventive measures implemented at the study site during the level 3 lockdown were effective in controlling both primary and secondary emissions. This finding aligns with Feng et al. (2021) in China. Besides, the OC/EC ratio is frequently utilized to estimate emissions of primary and secondary organic carbon from different sources (Zheng et al. 2019). A higher ratio typically indicates the prevalence of biomass burning emissions, while a lower ratio suggests a dominance of fossil fuel emissions (Ram et al. 2008; Saarikoski et al. 2008). For example, the ratio of 1.0 to 4.2 were reported for vehicles exhausts (Lough et al. 2007), 2.5 to 10.5 for coal combustion sources (Chen et al. 2006), and 3.8 to 13.2 for biomass burning (Koçak et al. 2021). Remarkably, in this study, all OC/EC ratios in both PM_1.0_ and PM_2.5_ consistently surpassed 2.0 before and during the pandemic, implying the formation of secondary organic carbon (Huang et al. 2021; Xu et al. 2018). These findings were further corroborated by studies conducted by Feng et al. (2021) in China and Chatterjee et al. (2021) in India.

Table S7 shows the seasonal variations of OC and EC levels in PM_1.0_ and PM_2.5_ before and during the pandemic. The highest mean values of both OC and EC in PM_1.0_ and PM_2.5_ were observed during winter (PM_1.0_: 2.58, 1.32 μg/m^3^ and PM_2.5_: 3.09, 1.48 μg/m^3^) and autumn (PM_1.0_: 2.30, 1.05 μg/m^3^ and PM_2.5_: 2.90, 1.29 μg/m^3^), followed by spring (PM_1.0_: 1.74, 0.52 μg/m^3^ and PM_2.5_: 1.84, 0.53 μg/m^3^) and summer (PM_1.0_: 1.71, 0.50 μg/m^3^ and PM_2.5_: 2.04, 0.55 μg/m^3^), demonstrating a high rate of emissions or meteorological effects (wind speed, temperature, relative humidity, wind direction etc.) (Sharma et al. 2022), which are reliable with earlier studies (Zhang et al. 2016; Kim et al. 2021). During the autumn and winter seasons, the highest OC and EC levels might be correlated with local pollution sources (vehicle emissions and coal combustion), attributed to the relaxation of PAL2 lockdown regulations. In addition, during winter, elevated levels of OC and EC can also be attributed to the influence of monsoons and haze originating from Asia (Fang et al. 2007). Despite this, spring and summer indicated a remarkable dip in both OC and EC values, indicating the significant impact of the PAL3 lockdown in reducing primary emissions at the study site. In addition, the highest OC/EC ratio in both PM_1.0_ and PM_2.5_ in spring and summer can be attributed to the formation of secondary organic carbon via photochemical oxidation (Li et al. 2020).

Table S8 indicates the mean Benzo[a]pyrene equivalent (BaPeq) levels in both PM_1.0_ and PM_2.5_ before and during the pandemic outbreak. Before PAL3, the mean values of BaPeq in PM_1.0_ and PM_2.5_ were 0.871 ± 0.478 ng BaPeq/m^3^ and 1.01 ± 0.254 ng BaPeq/m^3^ respectively, which were 2.2 to 2.8 times higher than the values during the pandemic alert (0.304 ± 0.029 ng BaPeq/m^3^ and 0.463 ± 0.172 ng BaPeq/m^3^). These results illustrate that the restrictions implemented to address COVID-19 had a positive impact on air quality and mitigated health risks associated with PAH exposure (Liu et al. 2022). Besides, Table S9 displays the values of BaPeq-PAHs in PM_1.0_ and PM_2.5_, followed the order winter > autumn > summer > spring. This seasonal pattern aligns with observations by Guzmán et al. (2023) in Spain. During winter, the elevated BaPeq-PAHs values may be attributed to variations in meteorological conditions (Tan et al. 2006), such as high relative humidity (RH > 70%) and low wind speed (WS < 0.1 m/s) (Kuo et al. 2008; Hsu and Cheng 2019). Additionally, during winter, the monsoonal winds transport pollutants from Mongolia and continental regions to the study site, resulted in higher BaPeq-PAHs levels (Chi et al. 2017). Also, the relaxation of pandemic restrictions during winter and autumn may also lead to an increase in BaPeq levels. Nevertheless, in spring, a remarkable dip of in BaPeq concentrations was noticed in both PM_1.0_ and PM_2.5_ due to the stoppage of industrial and anthropogenic emissions consequent upon stringent lockdowns.

### Source apportionment

#### Sulfur Oxidation Ratio (SOR) and Nitrogen Oxidation Ratio (NOR)

In the present study, SOR and NOR were used to investigate sulfate and nitrate formation. This method is very useful for converting primary pollutants (SO_2_ and NO_2_) into secondary ions (SO_4_^2-^ and NO_3_^-^) (Gao et al. 2021). Its calculation method is given below in eq. (10) and eq. (11).

10 $$\boldsymbol{SOR}=\frac{\boldsymbol{nss}-{\boldsymbol{SO}}_{\textbf{4}}^{\textbf{2}-}}{\boldsymbol{nss}-{\boldsymbol{SO}}_{\textbf{4}}^{\textbf{2}-}+{\boldsymbol{SO}}_{\textbf{2}}}$$

11 $$\boldsymbol{NOR}=\frac{{\boldsymbol{NO}}_{\textbf{3}}^{-}}{{\boldsymbol{NO}}_{\textbf{3}}^{-}+{\boldsymbol{NO}}_{\textbf{2}}}$$

Where nss-SO_4_^2-^ is the non-sea salt sulfate concentration ([SO_4_^2-^]- 0.25 x [Na^+^]), NO_3_^-^ is the concentration of nitrate, while SO_2_ and NO_2_ are the concentration data measured by the atmospheric stations.

Based on Table S10, the average SOR values for PM_1.0_ and PM_2.5_ showed a incredible increase during the pandemic, with values of 0.629 ± 0.076 and 0.636 ± 0.075 respectively, compared to pre-pandemic values of 0.283 ± 0.090 and 0.299 ± 0.111. In contrast, there was a slight decrease in the average NOR values during the pandemic, with values of 0.066 ± 0.015 and 0.085 ± 0.023 compared to pre-pandemic values of 0.078 ± 0.059 and 0.097 ± 0.081. According to Gao et al. (2021), a SOR value above 0.25 indicates secondary inorganic aerosol (SIA), while a NOR value below 0.10 suggests primary pollutants (Ohta and Okita 1990). Remarkably, the present study noted a significant SOR value (>0.25) both before and during the PAL3 lockdown, implying the formation of secondary aerosols. These findings are supported by Huang et al. (2023) and Lin (2002) in Taiwan. Prior to the pandemic, higher SOR levels at the study site were associated with increased local emissions. However, during PAL3, the elevated SOR values were primarily attributed to ongoing coal power plant and semiconductor industry operations at the study location. Additionally, increased electricity usage resulting from individuals staying at home and relying on air conditioning during PAL3 may also contribute to higher SOR levels during the pandemic. According to (Tsai 2021), there was a significant 5.1% surge in residential electricity usage in Taiwan during the lockdown compared to pre-lockdown. Conversely, the reduction in NOR values in PAL3 seems to indicate some role of lockdown in hindering the formation of reduced primary aerosol levels within the study site. Furthermore, the SOR displayed seasonal fluctuations, with the highest values in spring (PM_1.0_: 0.629 ± 0.076 and PM_2.5_: 0.636 ± 0.075), and the lowest values in winter (PM_1.0_: 0.342 ± 0.078 and PM_2.5_: 0.353 ± 0.078) compared to the other seasons (Table S11), attributed to the temperature variation (30.8°C in spring and 19.2°C in winter, respectively). Zhang et al. (2011) observed that warm and humid conditions in summer and autumn lead to the creation of secondary particles containing high levels of sulfate. Similarly, Shen et al. (2008) discovered a positive correlation between higher temperatures and increased sulfur oxidation. Throughout all seasons, the NOR ratios consistently remained lower than the SOR ratios. The highest NOR values were observed during spring (PM_1.0_: 0.066 ± 0.015 and PM_2.5_: 0.085 ± 0.023), while the lowest values were recorded in winter (PM_1.0_: 0.027 ± 0.027 and PM_2.5_: 0.036 ± 0.018), which can be attributed to meteorological factors. Hu et al. (2014) found similar phenomena for NOR among different seasons.

#### Positive matrix factorization

According to PMF analysis, PM_1.0_-bound PAHs were determined to be influenced by two main factors: coal combustion and traffic emissions. On the other hand, PM_2.5_-bound PAHs were found to be influenced by three factors: coal combustion, traffic emissions, and steel sinter plants. More detailed descriptions of each source can be found below.

In PM_1.0_-bound PAHs (Fig. S1), Factor 1 loaded 47% and consisted of BghiP, Bbf, Pyr, Bap, IND, and FL. According to Wang et al. (2020), Bbf, and Pyr are linked with coal combustion. Therefore, this factor may be associated with coal-fired boilers. Factor 2 loaded 53%, with a high load of Nap and Flu. Nap has been related with gasoline-powered sources (Guo et al. 2003; Fang et al. 2004), indicating a possible association with traffic emissions.

However, in PM_2.5_-bound PAHs (Fig. S2), Factor 1 accounted for 28% and was largely comprised of BghiP, PA and Acp. According to Wang et al. (2020), BghiP and PA are commonly found in coal combustion. Additionally, Factor 2 was loaded at 50% and showed a significant correlation with Nap and Flu. Nap primarily relates to gasoline-powered sources (Fang et al. 2004; Guo et al. 2003). Therefore, this factor may be linked to traffic emissions. In addition, Factor 3 accounted for 22.1% and mainly consisted of AcPy. Pyr indicate the presence of iron and steel sinter plants (Chen et al. 2016). This suggests that this factor may be related to iron and steel sinter plants.

#### Potential source contribution function

Fig. S3a and Fig. S3b depict the proportion of potential sources of fine particles (PM_1.0_ and PM_2.5_) to the monitoring site. Prior to the pandemic, areas with higher PSCF values (> 0.6) were mainly located in Central and Southwestern Taiwan. Under stable atmospheric conditions, the influence of fine particles was primarily from nearby stationary pollution sources (thermal power plants and waste incineration plants, etc.) or traffic exhausts (such as emissions from cars, trucks, and other vehicles). However, during PAL3, there was a substantial decrease in the PSCF values due to the implementation of strict pandemic control measures, resulting in reduced local/regional pollution and traffic emissions. Additionally, during the pandemic, continental areas experienced the highest PSCF values, suggesting that the study site may still be impacted by the long-range transport (LRT) of fine aerosols.

Fig. S4 illustrates the cluster analysis of PM_1.0_ and PM_2.5_ air mass tracking trajectories over a 48h period during the seasonal sampling in central Taiwan. The study identified five distinct clusters, denoted as Cluster 1 (red color), Cluster 2 (brown color), Cluster 3 (green color), Cluster 4 (blue color), Cluster 5 (purple color) respectively. As a result, Cluster 1 represents 65.9% of the total trajectories of both PM_1.0_ and PM_2.5_, originating from Taiwan (local pollution). Cluster 2, encompassing trajectories from the southern to central regions of Taiwan, represents 60.2% of total trajectories. Cluster 3, accounts for 39.4% of the total, originated from the northeastern part of the Asian continent. Cluster 4, accounts for 23.9% of the total, initiates from Gobi desert in Mongolia and extended through Northern continental areas, carries significant levels of anthropogenic pollutants towards the study location through long-range transport. Cluster 5 (10.6% of total) from oceans (ships and small boats etc.). In summary, Cluster 1, originating from Taiwan, had the highest pollution levels among the five clusters. It exhibited the highest concentrations of PM and was strongly influenced by local pollution sources.

### Health risk assessment

**Fig. 5:**
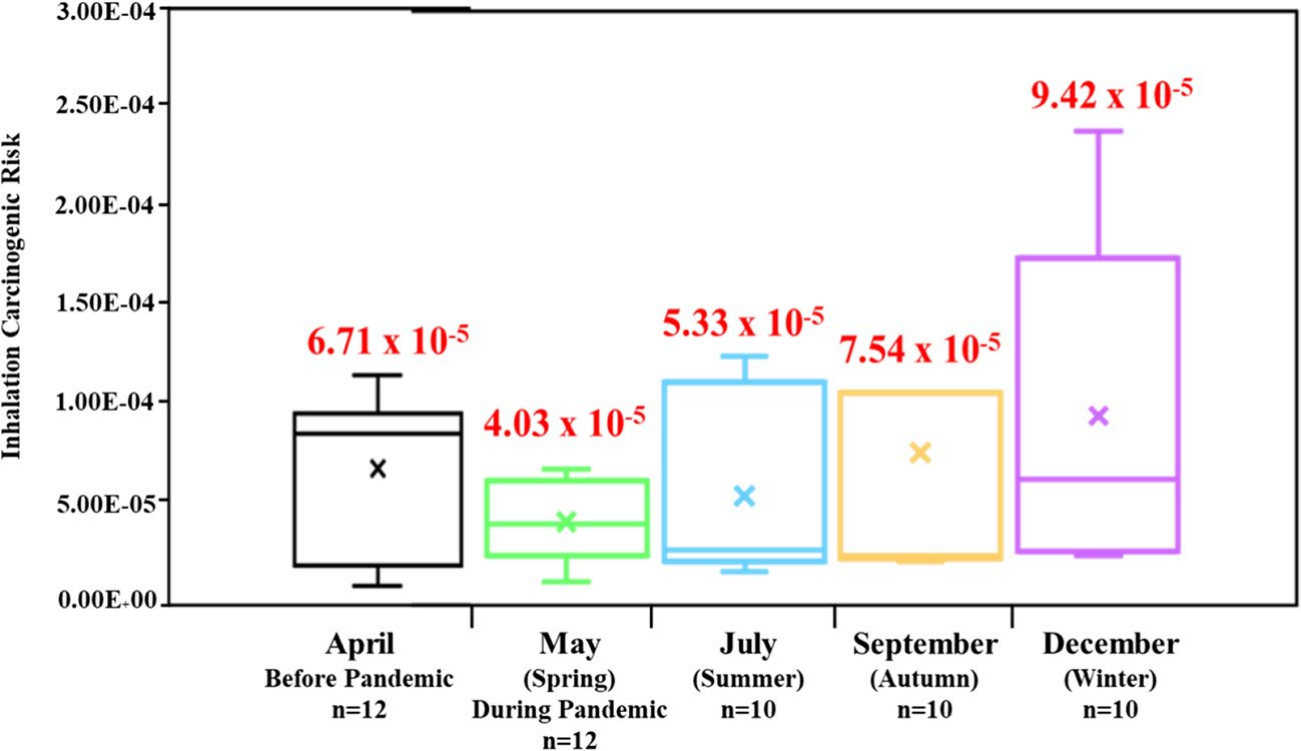
ILCR of PAHs in PM_2.5_ over different scenarios in 2021

Fig. 5 reveals the ILCR of PAHs in PM_2.5_ before PAL3 were higher 6.71 x 10^-5^ than 4.03 x 10^-5^ in PAL3. These results comply with the 10^-6^-10^-4^ standard established by the United States Environmental Protection Agency (USEPA 2011). Prior to the pandemic, it was estimated that the ILCR value of PAHs in PM_1.0_ contributed to 89% of PM_2.5_, while it accounted for 73% in PAL3, resulting in an average decrease of 51% and 40% respectively. This reduction indicates the successful enforcement of measures to reduce both stationary and vehicular emissions at the designated site during the pandemic outbreak. According to Liu et al. (2022), controlling coal combustion and vehicle emissions is an effective approach to mitigating health risks associated with PAHs. Similarly, Rabin et al. (2022) found a comparable situation in Bangladesh during COVID-19 restrictions. Furthermore, Fig. 5 reveals that the ILCR of PAHs in PM_2.5_ fluctuates by seasons with the sequence winter > autumn > summer > spring, with PM_1.0_ contributions of 73%, 69%, 79% and 67% respectively. During the winter, the relaxation of PAL2 regulations led to an increase in PM_2.5_ emissions from anthropogenic activities and long-range transport. This resulted in higher levels of BaPeq and an increased incremental lifetime cancer risk of 9.42 x 10^-5^. However, during spring, when the pandemic escalated to Level 3, there was a significant decline of 4.03 x 10^-5^ in ILCR. This decrease can be attributed to the strict control measures implemented, which effectively reduced industrial and anthropogenic emissions.

## Conclusions

The present study revealed higher values of both PM_1.0_ and PM_2.5_ before the COVID-19 Level 3 lockdown at Taichung, which notably decreased during the pandemic alert due to a reduction in industrial and anthropogenic emissions. According to PMF analysis, PM_1.0_-bound PAHs were determined to be influenced by two main factors: coal combustion and traffic emissions. On the other hand, PM_2.5_-bound PAHs were found to be influenced by three factors: coal combustion, traffic emissions, and steel sinter plants. Moreover, prior to the pandemic, SO_4_^2-^ (25.5%), NO_3_^-^ (22.7%) and Na^+^ (12.7%) were the main components of PM_1.0_, whereas SO_4_^2-^ (28.1%), NO_3_^-^ (29.4%) and NH_4_^+^ (10.1%) prevailed in PM_2.5_. In addition, PM_1.0_ shows a greater contribution to total PM_2.5_ levels in all seasons, with a ratio larger than 0.5. It is worth noting that the SOR value was higher (> 0.25) in both pre and post pandemic periods, likely due to stationary sources and long-range transport. Besides, during the pandemic alert, there was a considerable decrease in vehicle emissions leading to a sharp decline of NO_3_^-^ in both PM_1.0_ (9.6%) and PM_2.5_ (12.5%). Furthermore, the ILCR values of PAHs in PM_1.0_ and PM_2.5_ decreased significantly in PAL3, with an average reduction of 51% and 40%, respectively, largely attributed to a reduction in industrial and anthropogenic emissions. Finally, the difference in the Level 2 alert and Level 3 warning of the COVID-19 outbreak improves our understanding of changes PAHs in personal exposure to in PM_2.5_ and source contribution by PM_1.0_ resultant health risks through prospective assessments.

## Supplementary information

ESM 1 (PDF 426 kb)

## Data Availability

Supporting data is provided in the manuscript.
