# Supplementary material for: A comprehensive examination of temporal-seasonal variations of PM1.0 and PM2.5 in taiwan before and during the COVID-19 lockdown
Source: Environ Sci Pollut Res Int. 2024 Apr 18;31(21):31511–23. doi: 10.1007/s11356-024-33174-4 (PMC11711775; doi:10.1007/s11356-024-33174-4)
Supplement: Supplementary file 1 — (PDF 426 kb) [file 11356_2024_33174_MOESM1_ESM.pdf]

Supporting information for:

A Comprehensive Examination of Temporal-seasonal Variations of $PM_{1.0}$ and $PM_{2.5}$ in Taiwan Before and During the COVID-19 Lockdown

Shahzada Amani Room [1], Yi Chen Chiu [1], Shih Yu Pan[1], Yu-Cheng Chen[2], Ta-Chih Hsiao[3], Charles C.-K. Chou[4], Majid Hussain[5] and Kai Hsien Chi [1 2*]

[1] *Institute of Environmental and Occupational Health Sciences, National Yang Ming Chiao Tung University, Taipei 112, Taiwan*

[2] *National Institute of Environmental Health Sciences, National Health Research Institutes, 35 Keyan Road, Zhunan Town, Miaoli, Taiwan*

[3] *Graduate Institute of Environmental Engineering, National Taiwan University, Taiwan*

[4] *Research Center for Environmental Changes, Academia Sinica, Taipei 115, Taiwan*

[5] *Department of Forestry and Wildlife Management, University of Haripur, 22620, Hattar Road, Haripur City, KP, Pakistan*

[*]Corresponding author. Tel: +886-2-2826-7352

E-mail address: khchi@nycu.edu.tw

Text S1

PAHs analysis

The samples were first chopped into small pieces and then soaked in 700 ml of dichloromethane. Subsequently, Soxhlet extraction was carried out followed by refluxing at a flowrate of 3 cycles/hour for a total duration of 16 hrs. The extraction was conducted under a 4:1 (dichloromethane/n-hexane) solvent mixture. The extract was further reduced to approximately 2 milliliters by employing a rotary evaporator that was coupled with a constant temperature bath (with temperature set at 30 ± 1°C). After extraction, the extracted solvent was purified using an activated carbon column and analyzed with a Thermo Scientific™ TSQ 8000 Evo Triple Quadrupole GC-MS/MS. This study indicated 27 different types of polycyclic aromatic hydrocarbons.

Water-soluble ions

A total of ten water-soluble ionic species, including Cl^-, $SO4^{2-}$, $NO3^-$, NH_4^+, $NO2^-$, $PO4^{3-}$, Na^+, K^+, Mg^{2+} and Ca^{2+}, were extracted from the filter samples using sonication. After extraction, the filters were put into a plastic bottle and added 10 milliliters of deionized water (impedance value > 18.0 MΩ/cm), The bottle was securely sealed and placed in an ultrasonic shaker for 90 minutes to guarantee water stability. Subsequently, the extracts were filled up into 10 milliliter pre-filled syringes with pore size of 0.2 µm and a diameter of 20 millimeters. Finally, the filtration was then carried out on a Dionex™ ICS-1000 (Thermo Fisher Scientific Ion Chromatograph) for analysis.

Carbonaceous Species

A 0.210 cm^2 filter sample was initially put through carbonaceous (OC/EC) analysis by placing it into a contaminant-free helium chamber. This was then followed by gradually raising the temperature of the chamber at intervals of 120, 250, 450, and 500°C to isolate CO_2 from the filters and thus attain organic constituents (OC1, OC2, OC3, OC4). Afterward, the samples were placed in a helium chamber containing 2% oxygen at temperatures of 500°C, 700°C, and 800°C to obtain ECs consisting of EC1, EC2, and EC3. The inlet temperature was increased, resulting in the formation of some optical pyrolytic carbon (OPC) by reflecting the 633 nm light from the helium and neon lasers.

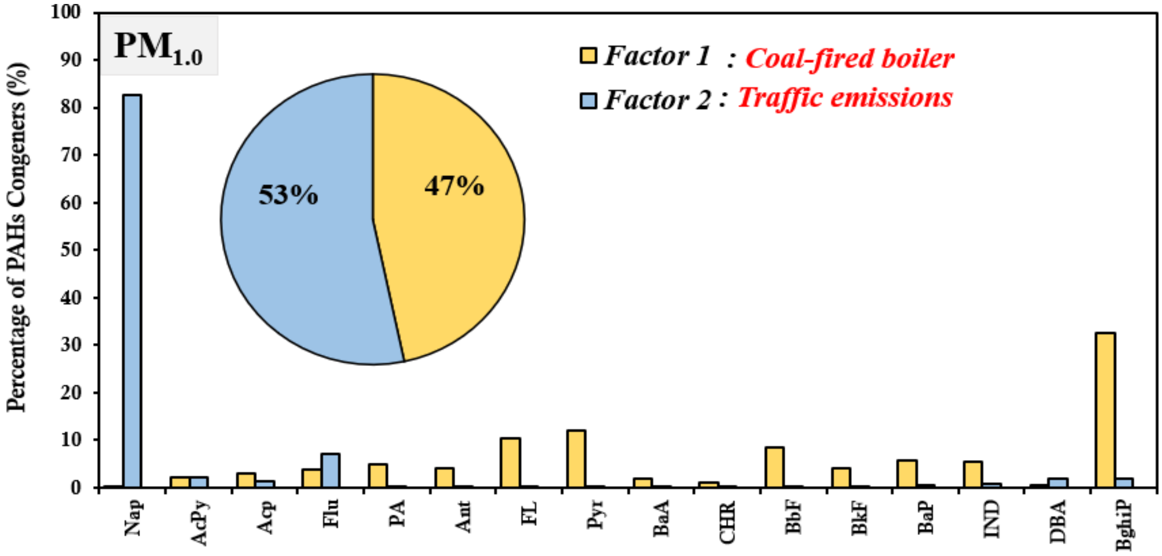

Fig. S1. PMF analysis of PAHs in $PM_{1.0}$ during the sampling period of 2021

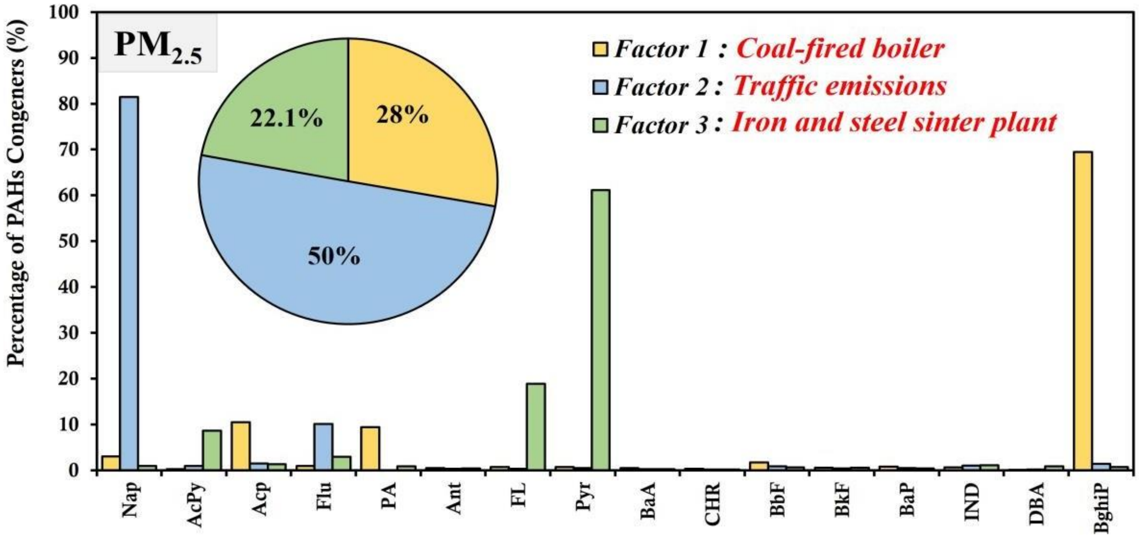

Fig. S2 PMF analysis of PAHs in $PM_{2.5}$ during the sampling period of 2021

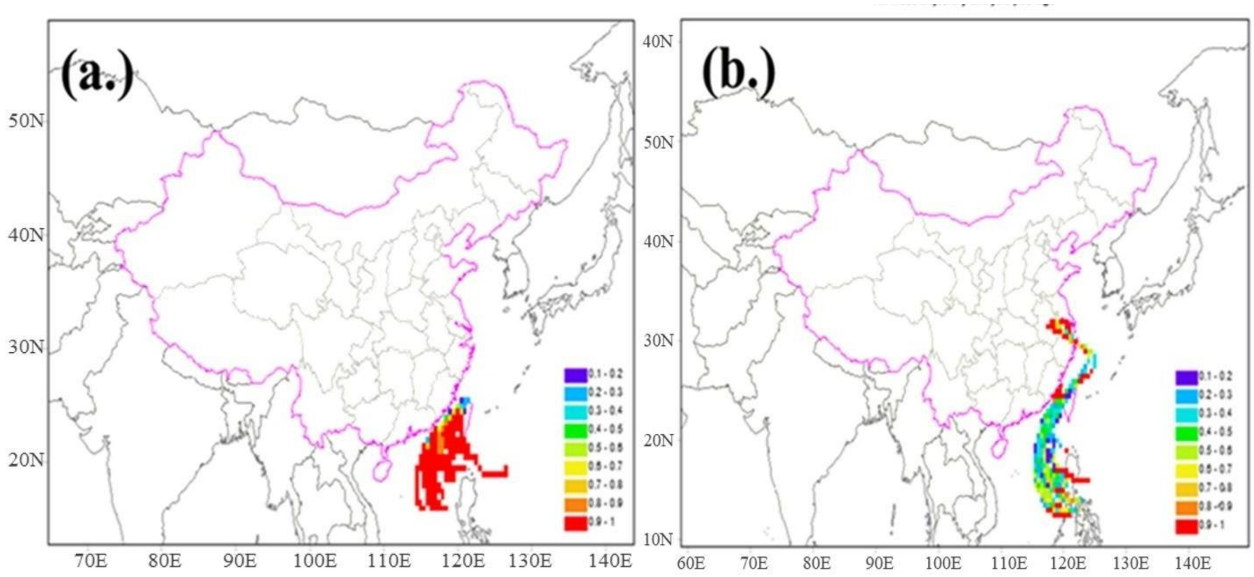

Fig. S3. The result of PSCF analysis of $PM_{1.0}$ and $PM_{2.5}$ before and during epidemic alert
a. April (before pandemic), b. May (during pandemic)

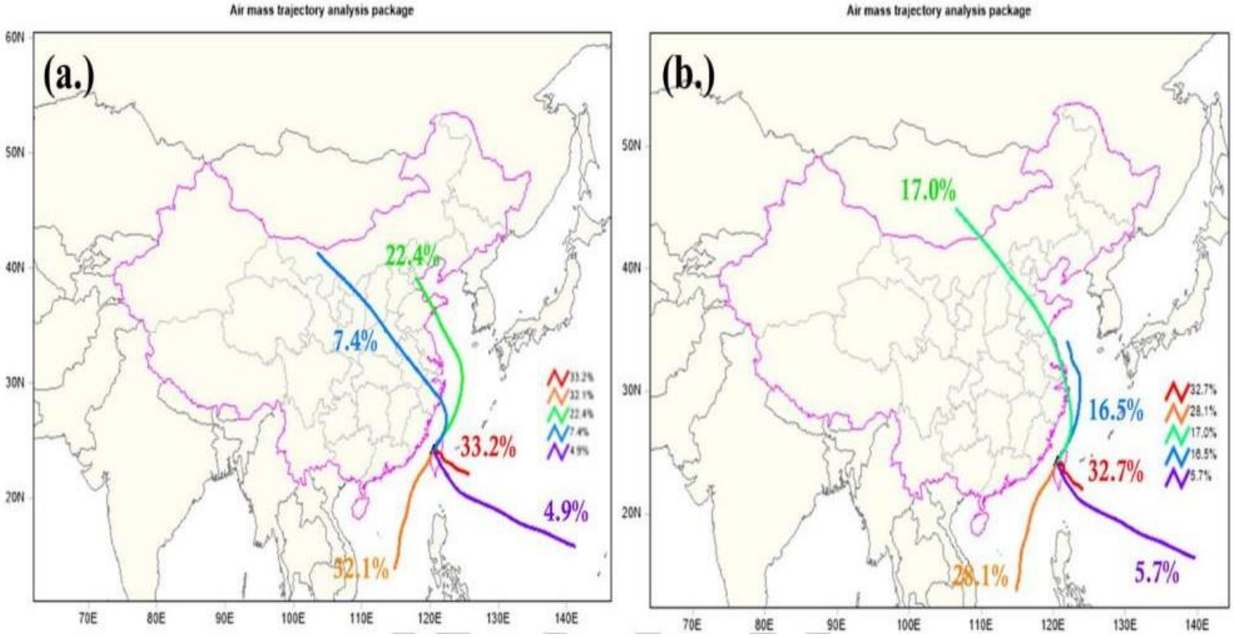

Fig. S4. Cluster analysis of air mass backtracking at internal stations over different seasons (a. $PM_{1.0}$, b. $PM_{2.5}$)

Tab S1. The Benzo[a]pyrene of toxic equivalency factors of individual species of PAHs (BaP-TEF_i)

27 Compounds of PAHs	Abb.	TEF
**Naphthalene**	Nap	0.001
**Acenaphthylene**	AcPy	0.001
**Acenaphthene**	Acp	0.001
**Flourene**	Flu	0.001
**Phenanthrene**	PA	0.001
**Fluoranthene**	FL	0.001
**Pyrene**	Pyr	0.001
**2-Methylnaphthalene**	2-MN	0.001
**Perylene**	PER	0.001
**Chrysene**	CHR	0.01
**Benzo[g,h,i]perylene**	BghiP	0.01
**Anthracene**	Ant	0.01
**Benzo[e]pyrene**	BeP	0.01
**Indeno[1,2,3-cd]pyrene**	IND	0.1
**Benzo[b]fluoranthene**	BbF	0.1
**Benzo[k]fluoranthene**	BkF	0.1
**Benzo[a]anthracene**	BaA	0.1
**Cyclopenta[c,d]pyrene**	CYC	0.1
**Benzo[j]fluoranthene**	BjF	0.3
**Dibenzo[a,h]pyrene**	DBahP	0.3
**Dibenzo[a,e]pyrene**	DBP	0.4
**Dibenzo [a, i]pyrene**	DBaiP	0.6
**Dibenzo[a,h]anthracene**	DBA	1.0
**Benzo[a]pyrene**	BaP	1.0
**5-Methylchrysene**	5-MC	1.0
**Benzo[c]fluorene**	BcFE	20
**Dibenzo[a,l]pyrene**	DBalP	30

Table S2. Concentration of $PM_{1.0}$ and $PM_{2.5}$ before and during the epidemic alert

	**Concentration**		**Ratio**	**Timing Line**
	**$PM_{1.0}$**	**$PM_{2.5}$**		
**Before Pandemic Alert**	16.6 ± 6.41	20.9 ± 6.92	0.79	April (n=12)
**During the Level 3 Alert**	13.0 ± 2.36	15.3 ± 2.51	0.85	May (n=12)

# Unit: concentration = μg/m^3

Table S3. Concentration of $PM_{1.0}$ and $PM_{2.5}$ over different seasons.

	**Concentration**		**Ratio**
	**$PM_{1.0}$**	**$PM_{2.5}$**	
**Spring**	13.0 ± 2.36 (n=12)	15.3 ± 2.51 (n=12)	0.85
**Summer**	13.9 ± 3.10 (n=10)	15.7 ± 2.64 (n=10)	0.88
**Autumn**	17.8 ± 3.78 (n=10)	25.5 ± 2.42 (n=10)	0.70
**Winter**	19.7 ± 5.08 (n=10)	27.1 ± 7.90 (n=10)	0.73

# Unit: concentration = μg/m^3

Tab S4. Concentrations of WSIs in $PM_{1.0}$ and $PM_{2.5}$ before and during the epidemic alert

		$PM_{1.0}$		$PM_{2.5}$		Ratio
		Mean	SD	Mean	SD	
Conc.	April (n=12)	**12.0**	3.93	**12.1**	6.85	0.99
	May (n=12)	**10.3**	1.83	**10.4**	1.95	0.99
	p-value	0.522		0.873		---

# Unit: concentration = μg/m^3

# p-value：Mann-Whitney U test (*=p-value<0.05)

Tab S5. Concentrations of WSIs in $PM_{1.0}$ and $PM_{2.5}$ in different seasons.

		$PM_{1.0}$		$PM_{2.5}$		Ratio
		**Mean**	**SD**	**Mean**	**SD**	
**Conc.**	**Spring (n=12)**	**10.3**	1.83	**10.4**	1.95	0.99
	**Summer (n=10)**	**6.72**	0.57	**7.26**	0.42	0.90
	**Autumn (n=10)**	**8.43**	4.39	**9.88**	2.59	0.85
	**Winter (n=10)**	**4.82**	2.73	**5.45**	2.57	0.88

# Unit: concentration = μg/m^3

Tab S6. Concentration of OC and EC in $PM_{1.0}$ and $PM_{2.5}$ before and during the epidemic alert

		$PM_{1.0}$			$PM_{2.5}$			Ratio
		**Mean**	**SD**	**Range**	**Mean**	**SD**	**Range**	
**April (n=12)**	**OC**	**3.53**	0.118	3.46-3.62	**3.79**	0.031	3.77-3.81	0.93
	**EC**	**1.05**	0.147	0.903-1.15	**1.12**	0.201	0.919-1.26	0.94
	**TC**	**4.58**	0.029	4.56-4.60	**4.91**	0.231	4.75-5.07	0.93
	**OC/EC**	**3.42**	0.593	3.00-3.83	**3.43**	0.587	3.02-3.85	---
**May (n=12)**	**OC**	**1.74**	0.350	1.38-2.33	**1.84**	0.456	1.39-2.63	0.95
	**EC**	**0.52**	0.082	0.437-0.601	**0.53**	0.072	0.458-0.602	0.98
	**TC**	**2.26**	0.383	1.81-2.91	**2.36**	0.507	1.87-3.26	0.96
	**OC/EC**	**3.39**	0.634	2.41-4.11	**3.46**	0.650	2.72-4.22	---

# Unit: Concentration = $\mu g/m^3$

Tab S7. Concentrations of OC and EC in $PM_{1.0}$ and $PM_{2.5}$ in different seasons.

		$PM_{1.0}$		$PM_{2.5}$		Ratio
		Mean	SD	Mean	SD	
**Spring (n=12)**	**OC**	**1.74**	0.350	**1.84**	0.456	0.95
	**EC**	**0.52**	0.082	**0.53**	0.072	0.98
	**TC**	**2.26**	0.383	**2.36**	0.507	0.96
	**OC/EC**	**3.39**	0.634	**3.46**	0.650	---
**Summer (n=4)**	**OC**	**1.71**	0.504	**2.04**	0.627	0.84
	**EC**	**0.50**	0.089	**0.55**	0.085	0.91
	**TC**	**2.21**	0.593	**2.59**	0.712	0.85
	**OC/EC**	**3.36**	0.408	**3.65**	0.574	---
**Autumn (n=4)**	**OC**	**2.30**	0.282	**2.90**	0.313	0.79
	**EC**	**1.05**	0.211	**1.29**	0.106	0.81
	**TC**	**3.35**	0.493	**4.19**	0.207	0.80
	**OC/EC**	**2.21**	0.175	**2.27**	0.428	---
**Winter (n=4)**	**OC**	**2.58**	**1.01**	**3.09**	0.893	0.83
	**EC**	**1.32**	**0.225**	**1.48**	0.214	0.89
	**TC**	**3.90**	**1.23**	**4.57**	1.11	0.85
	**OC/EC**	**1.91**	**0.437**	**2.06**	0.305	---

# Unit: concentration = μg/m^3

Table S8. Concentration of BaPeq-PAHs in $PM_{1.0}$ and $PM_{2.5}$ before and during the epidemic

		$PM_{1.0}$		$PM_{2.5}$		Ratio
		Mean	SD	Mean	SD	
**BaPeq-PAHs Conc.**	**April (n=12)**	**0.871**	0.478	**1.01**	0.254	0.86
	**May (n=12)**	**0.304**	0.029	**0.463**	0.172	0.66
	**p-value**	0.078		0.262		---

# Unit: BaPeq-PAHs concentration = ng BaPeq/m^3

# p-value：Mann-Whitney U test (*=p-value<0.05)

Table S9. Concentration of BaPeq-PAHs in $PM_{1.0}$ and $PM_{2.5}$ in different seasons.

		$PM_{1.0}$		$PM_{2.5}$		Ratio
		**Mean**	**SD**	**Mean**	**SD**	
**BaPeq-PAHs**	**Spring (n=12)**	**0.304**	0.029	**0.463**	0.172	0.66
	**Summer (n=10)**	**0.422**	0.276	**0.612**	0.513	0.69
**Conc.**	**Autumn (n=10)**	**0.681**	0.685	**0.867**	1.33	0.79
	**Winter (n=10)**	**0.722**	0.173	**1.08**	0.898	0.67

Unit: BaPeq-PAHs concentration = ng BaPeq/m^3

Table S10 S.O.R. and N.O.R. ratios before and during epidemic alert

	**$PM_{1.0}$**		**$PM_{2.5}$**	
	**SOR**	**NOR**	**SOR**	**NOR**
**April (n=12)**	0.283 ± 0.090	0.078 ± 0.059	0.299 ± 0.111	0.097 ± 0.081
**May (n=12)**	0.629 ± 0.076	0.066 ± 0.015	0.636 ± 0.075	0.085 ± 0.023

Table S11. S.O.R. and N.O.R. ratios in different seasons.

	$PM_{1.0}$		$PM_{2.5}$	
	**SOR**	**NOR**	**SOR**	**NOR**
**Spring (n=12)**	0.629 ± 0.076	0.066 ± 0.015	0.636 ± 0.075	0.085 ± 0.023
**Summer (n=10)**	0.354 ± 0.064	0.050 ± 0.019	0.358 ± 0.068	0.072 ± 0.030
**Autumn (n=10)**	0.463 ± 0.277	0.039 ± 0.020	0.564 ± 0.180	0.041 ± 0.023
**Winter (n=10)**	0.342 ± 0.078	0.027 ± 0.027	0.353 ± 0.078	0.036 ± 0.018
